# Analysis of Soybean Somatic Embryogenesis Using Chromosome Segment Substitution Lines and Transcriptome Sequencing

**DOI:** 10.3390/genes10110943

**Published:** 2019-11-19

**Authors:** Si-Nan Li, Peng Cheng, Yun-Qi Bai, Yan Shi, Jing-Yao Yu, Rui-Chao Li, Run-Nan Zhou, Zhan-Guo Zhang, Xiao-Xia Wu, Qing-Shan Chen

**Affiliations:** College of Agriculture, Northeast Agricultural University, Harbin 150030, Heilongjiang, China; snli@neau.edu.cn (S.-N.L.); naturechengpeng@163.com (P.C.); kldbyq123@163.com (Y.-Q.B.); llowkeyy@126.com (Y.S.); Jingyaofish@163.com (J.-Y.Y.); liruichao2018@126.com (R.-C.L.); zrn19940615@163.com (R.-N.Z.)

**Keywords:** soybean, somatic embryogenesis, CSSL, transcriptome sequencing, RT-qPCR, haplotype analysis

## Abstract

Soybean is an important cash crop that is widely used as a source of vegetable protein and edible oil. The regeneration ability of soybean directly affects the application of biotechnology. In this study, we used the exogenous hormone 2,4-D to treat immature embryos. Different levels of somatic incidence were selected from the chromosome segment substitution lines (CSSLs) constructed by SN14 and ZYD00006. Transcriptome sequencing of extreme materials was performed, and 2666 differentially expressed genes were obtained. At the same time, a difference table was generated by combining the data on CSSL rearrangement. In the extreme materials, a total of 93 differentially expressed genes were predicted and were then analyzed by cluster analysis and Gene Ontology (GO) annotation. After screening and annotating the target genes, three differentially expressed genes with hormone pathways were identified. The expression patterns of the target genes were verified by real-time quantitative PCR (qRT-PCR). Haplotype polymorphism detection and linkage disequilibrium analysis were performed on the candidate gene *Glyma.09g248200*. This study provided more information on the regulation network of soybean somatic embryogenesis and regeneration processes, and further identified important genes in the soybean regeneration process and provided a theoretical basis for accelerating the application of biotechnology to soybean for improving its breeding efficiency.

## 1. Introduction

Soybean is an important food and oil crop grown all over the world [1]. In order to accelerate the soybean breeding process, the application of biotechnology is indispensable. Due to the long genetic transformation cycle of soybean and its low regeneration efficiency, research on soybean gene function is relatively lagging in comparison to other plants. Therefore, revealing the mechanism of soybean regeneration is of particular importance for studying the function of soybean genes and accelerating their breeding process.

On the 125th anniversary of its publication, *Science* magazine asked, “How can a single somatic cell form a complete plant?” with the expectation that people would analyze the principle of this phenomenon in future research [2,3]. The process of a single cell developing into a complete plant involves the process of regeneration, which refers to the phenomenon by which plant cells can develop into normal and complete plants, in vitro, under certain culture conditions. The regeneration ability of different plants varies greatly, and the regeneration ability can also differ by the variety and among different explants of the same plant. How to improve the regeneration ability of soybean and facilitate efficient and wide use of biotechnology for soybean was the main basis for our research.

Soybean somatic embryos can be induced by treating immature embryos with hormones. Somatic embryos can be used to induce new plants on a large scale or to study cell differentiation, gene expression, and genetics. They are among the most powerful tools for soybean biotechnology research. Therefore, the induction rate of somatic embryos in soybean is the key to solving the problem of soybean regeneration.

Previous studies have applied molecular marker technology to the mapping of quantitative trait loci (QTLs) of somatic embryogenesis in soybean. Song et al. constructed BC_5_F_6_ recombinant inbred lines from high-regenerated and low-regenerated soybean materials. A total of 126 inbred lines were obtained, and simple sequence repeat (SSR) molecular markers closely related to somatic embryogenesis were identified by mapping the QTLs [4]. Yang et al. studied the effect of an immature embryo size on the somatic embryo induction rate. The results showed that an immature embryo size, i.e., 4–5 mm, was associated with the highest immature embryo incidence. The callus and somatic embryo induction rates of 184 recombinant inbred lines, derived from Kefeng 1 *Nannong 1138-2, were used to evaluate the tissue culture of soybean. This ability was used as an indicator to locate the QTLs. Seven related QTLs were detected in the two indicators. The results showed that the alleles in Kefeng 1 could improve the rate of embryogenesis and the embryogenesis of immature embryos [5].

With the further development of sequencing technology, it is particularly important to study the phenotypic differences of plants using this technology. Zhang et al. considered *Glycine soja* to be a soybean wild progenitor, with much higher levels of genetic diversity compared to cultivated soybeans. The transcriptomes of the resistant and susceptible genotypes of wild soybean, *Glycine soja* Sieb and Zucc, were sequenced to examine the genetic basis of soybean cyst nematode (SCN) resistance, and large amounts of data were obtained. Further analysis of this data would be helpful in improving our understanding of the molecular mechanisms of soybean–SCN interaction and to facilitate the development of diverse SCN resistance cultivars [6]. Wang et al. used transcriptome sequencing technology to study the transcription factors in the adventitious shoot regeneration of *Arabidopsis thaliana* and identified 155 transcription factors [7]. Dorian et al. analyzed the transposons of the wild-type and *lore1* retrotransposon mutagenesis of *Lotus japonicus* by transcriptome sequencing and identified 231 differentially expressed genes [8].

At the same time, several genes related to soybean regeneration have been cloned or studied. Zhang et al. cloned the gene *GmESR1,* related to soybean regeneration. The functional analysis confirmed that this gene promoted soybean regeneration [9]. Zhou et al. cloned the soybean regeneration-related gene *GmWUS,* indicating that it participates in the differentiation and regeneration of cluster buds and improves the regeneration ability of soybean [10]. Jin et al. cloned the *GmLEC1-A* gene to explore its role in the soybean regeneration system under abscisic acid (ABA) treatment [11].

Soybean regeneration is limited by the soybean genotype. Some soybean varieties, such as Dongnong 50 and Tianlong 1, are the main acceptor materials for biotechnological applications. The main characteristics of soybean varieties are strong wild character and good regeneration. Through these receptors, germplasm with good characteristics can be obtained and transformed into the existing cultivated varieties by means of hybridization. The breeding time can be shortened by hybridization, but the target gene segments are easily lost in the process of hybridization, which hinders research on the gene function and molecular design breeding of soybean. The poor regeneration ability of cultivated soybean varieties has become a bottleneck in the application of soybean biotechnology. How to break the bottleneck of soybean regeneration being limited by the soybean genotype and make possible the direct application of soybean gene editing, molecular design breeding, and transgenic technology to the main varieties of soybean constitute the main problem to be solved.

The key to ensuring that biotechnology is widely used in the cultivation of soybean is to analyze the mechanism of soybean regeneration. This will allow the bottleneck problem of soybean regeneration being limited by the soybean genotype to be resolved. As a consequence, we would be able to identify the key genes related to soybean regeneration from the perspective of genes and to analyze the function and mechanism of promoting soybean regeneration and its molecular regulatory network in order to address the efficiency of soybean regeneration and transformation from the root. Analysis of the bottleneck problem of soybean regeneration being restricted by the soybean genotype provides insights into the regeneration mechanism of soybean and even plants more generally, forming the theoretical basis for the development of techniques useful for research on soybean gene function and gene editing, as well as molecular design breeding. These developments could help in promoting the extensive application of molecular breeding in the cultivation and improvement of excellent varieties of soybean toward achieving the ultimate goal of accelerating the process of soybean breeding and improving the yield, quality, and competitiveness of soybean in the international market.

In this study, 208 populations of chromosome segment substitution lines (CSSLs)—obtained by combining the parent, Suinong 14, with the wild soybean, ZYD00006—were used as materials to induce somatic embryogenesis by culturing immature embryos of parents and populations. The data on somatic embryogenesis were analyzed, and extreme materials of somatic embryogenesis were obtained. Combining the transcriptome sequencing data of extreme materials with the chromosome substitution sequencing data of CSSL populations, differentially expressed genes affecting somatic embryogenesis were obtained, clustered, annotated, and analyzed. Real-time quantitative PCR (RT-qPCR) was used to analyze the expression of candidate genes, which verified the accuracy of the sequencing results. Finally, the haplotype analysis of differentially expressed candidate genes was carried out to rationalize the causes for the phenotypic changes of extreme materials in the population. These studies laid a foundation for determining the mechanism of somatic embryogenesis and regeneration in soybean.

## 2. Materials and Methods

### 2.1. Construction of the CSSL Population and Calculation of the Extreme Phenotype of Somatic Embryogenesis

Suinong 14 (provided by the Suihua Branch of the Heilongjiang Academy of Agricultural Sciences, Suihua, Heilongjiang, China) and the wild soybean, ZYD00006 (provided by Chinese Academy of Agricultural Sciences, Beijing, China), were used as parents in this experiment. Suinong 14 was used as a recurrent parent, and the wild bean, ZYD00006, was used as a donor parent, to construct a chromosome segment substitution line population (CSSL). By 2017, 208 CSSLs had been obtained through markup selection [12].

The parents and CSSL population were planted at Xiangyang Farm, Harbin, Heilongjiang Province (latitude 45°450 N, longitude 126°380 E). The immature embryos of Suinong 14, ZYD00006, and CSSL were sampled after 3–4 weeks of flowering, and the pods were collected at the third to fifth nodes of the plant. Immature embryos from different plants of the same cultivar (line) were sampled as biological replicates. Ten pods were taken from each of the 3 replicates. Samples of the same number were collected, and immature embryos and pods were quickly separated and frozen in liquid nitrogen, then stored in a refrigerator at −80 °C for transcriptome sequencing.

Young soybean pods were rinsed with water, disinfected with 20% sodium hypochlorite solution for 20 min, then rinsed with sterile water 3–5 times under sterile conditions to remove residual sodium hypochlorite solution on the surface of the pods. The immature embryos were taken out with tweezers, and the 2 cotyledons were separated and cultured in a solid induction medium. Six to 10 immature embryos were placed in each bottle of the medium, and 3 replicates were set for each variety. After about 20 days of inoculation, the number of immature embryos forming somatic embryos was counted, and the induction rate was calculated using the formula below.

Induction rate (%) = The total number of immature embryos/immature embryos induced from somatic embryos

The formulation of the solid induction medium was as follows: 4.34 g·L^−1^ MS powder (PhytoTech, M524, PhytoTechnology Laboratories, Lenexa, Kansas, MO, USA), 30 g·L^−1^ sucrose (Sigma, V900116, Sigma-Aldrich, Saint Louis, MO, USA), 4 mL·L^−1^ 2,4-D (PhytoTech, D309, PhytoTechnology Laboratories, Lenexa, Kansas, MO, USA), and 7 g·L^−1^ Gelrite (Sigma,·G1910, Sigma-Aldrich, Saint Louis, MO, USA). The medium was sterilized at 121 °C for 20 min and then separated into sterilized bottles.

In order to obtain characteristics related to the relative somatic embryo incidence that differed from the parent material, the somatic embryo incidence in different strains was studied. First, phenotypic data on the somatic embryo induction rate was fitted according to normal distribution. If the condition of normal distribution was satisfied, the mean value of the normal distribution plus 2 times the standard deviation (μ + 2σ) was used as the demarcation line for screening the high induction rate of somatic embryos, and the mean minus 2 times the standard deviation (μ − 2σ) was used as the demarcation line for the low induction rate of somatic embryos to obtain the extreme somatic embryogenesis materials.

### 2.2. Analysis of the Import Fragments of Extreme Advantage CSSL Population Materials

From 2014–2017, 208 CSSLs were constructed in our laboratory by hybridizing the cultivated soybean, Suinong 14, with the wild soybean, ZYD00006, and then backcrossing Suinong 14 as the recurrent parent. The obtained CSSL population was resequenced, and information on each CSSL import fragment was obtained. Marker bb was used as the genetic background, marker aa was used as the homozygous import fragment, and marker ab was used as the heterozygous import fragment. After comparison, the physical location was obtained for each fragment (block) introduced into the soybean genome. We obtained the block information on each of the extreme materials and counted the homozygous (aa) blocks introduced into 3 or more extreme dominant materials as effective import fragments for somatic embryogenesis. Subsequently, the reference genome physical location of soybean was compared in the Phytozome database (https://phytozome.jgi.doe.gov/pz/portal.html#!info?alias=Org_Gmax), and the genes contained in the import fragments were obtained for subsequent analysis.

### 2.3. RNA Extraction, Library Construction, and Sequencing Data Processing

The five extreme dominant materials were named A–E, and the 5 extreme inferior materials were named F–J, and the parent, Suinong 14, was named X. The materials stored at −80 °C were used as period 1 (A1, B1, C1, ..., X1), and the calluses obtained after 20 days of culturing served as period 2 (A2, B2, C2, ..., X2).

All materials were rapidly frozen in liquid nitrogen at a low temperature and then rapidly ground into powder. The RNA was extracted by TRIzol (Invitrogen, 15596-026, Carlsbad, CA, USA), and the quality of the RNA was detected using 1% agarose gel electrophoresis and NanoDrop instrumentation. The concentration and purity of the RNA were determined by NanoDrop. The OD260/280 was required to be between 1.8 and 2.2, and 28S/18S was not less than 1. An Agilent Bioanalyzer 2100 (Agilent Technologies, Santa Clara, CA, USA) was used to detect the RNA integrity. All sequencing samples were rated as A-level, meeting the requirements of the library building.

Sequencing libraries were constructed after qualified RNA detection. The soybean genome (*Glycine max*) was used as a reference sequence in read mapping [13]. The sequencing instrument was an Illumina HiSeq 4000 PE 150. (Reference genome link: http://genome.jgi.doe.gov/pages/dynamicOrganismDownload.jsf?Organism=Gmax).

After sequencing, the raw data were analyzed by quality control (QC). The joints, the reads with an N ratio higher than 10%, and the bases with Q ≤ 20 were removed as low-quality reads, and the high-quality clean read data were used for subsequent analysis.

The DESeq R package (http://bioconductor.org/packages/release/bioc/html/DESeq.html) was used to screen genes or transcripts with significant differences in expression under two different conditions. Genes with a log2 fold change ≥1 or ≤1 and *p* < 0.05 were regarded as differentially expressed genes, which served as the basis for screening differentially expressed genes in transcriptome sequencing.

### 2.4. Analysis of Differentially Expressed Genes in Import Fragments and Transcriptome Sequencing

In order to accurately identify the major genes affecting somatic embryogenesis in the CSSL population, we combined the differentially expressed genes, obtained by transcriptome sequencing, with the genes contained in the CSSL block. A Microsoft Access database was used to transfer the data on differentially expressed genes between the two groups. The common genes were found by the comparison function. After the data were derived, the upregulation and downregulation of gene expression based on transcriptome sequencing and the annotations of the KEGG database were analyzed. According to the expression level, MEV4.9.0 software (https://sourceforge.net/projects/mev-tm4/) was used to draw a heatmap of differentially expressed genes, and WEGO software (http://wego.genomics.org.cn/) was used to analyze the enrichment of differentially expressed genes in order to uncover the main pathways and key genes affecting somatic embryogenesis.

### 2.5. RT-qPCR Analysis of Candidate Genes

Differentially expressed candidate genes were selected based on the results of gene annotation for further verification of their expression in extreme materials. By comparing the annotations of functions of the differentially expressed genes in the database, we found that 3 key genes were involved in hormone regulation or regeneration and speculated that these genes might be involved in somatic embryogenesis.

Real-time quantitative PCR (RT-qPCR) primers were designed using Primer Premier v6.0 (http://www.premierbiosoft.com/primerdesign/index.html). The actin 4 gene of soybean was selected as the internal reference gene. The material RNA was extracted by TRIzol, and the genomic DNA was then removed by a 4× gDNA wiper mix (Vazyme, R223, Vazyme biotech, Nanjing, China). The RNA was retrieved as a single-strand cDNA by a 5× HiScript II qRT SuperMix II (Vazyme, R223, Vazyme biotech, Nanjing, China). The RT-qPCR program used a Light Cycler 480 system (Roche, Roche Diagnostics, Basel, Switzerland) and the 2× ChamQ Universal SYBR qPCR Master Mix Kit (Vazyme, Q711, Vazyme biotech, Nanjing, China). The RT-qPCR analysis was carried out based on 3 biological replicates. The relative expression of the candidate genes was calculated according to the following formula:

Relative expression = 2^∆∆*C*t^, {∆∆Ct = [Ct_2_(*Gm* target genes) − Ct_2_(ACTIN4)] − [Ct_1_(*Gm* target genes) − Ct_1_ (ACTIN4)]}

### 2.6. Haplotype Analysis of Candidate Genes

The CDS sequences of the candidate genes and promoter sequences of 3000 bp upstream were obtained from the Phytozome database (https://phytozome.jgi.doe.gov/pz/portal.html). The single-nucleotide polymorphism (SNP) sequence information of the candidate genes in the CSSL population was obtained by local BLAST (Basic Local Alignment Search Tool) alignment of the parents and resequencing data of 208 CSSLs.

Then, DnaSP 5.10 software was used to analyze the haplotype distribution of these SNP sequences in the CSSLs. Excellent haplotypes were screened for using 5% of the total CSSLs contained in the haplotype as the dividing line. The ANOVA of the candidate genotypes and phenotypes was carried out using SPSS 17.0 software in order to determine the impact of each haplotype on the phenotype.

Next, Haploview 4.2 software (https://haploview.software.informer.com/4.2/) was used to analyze the loci of candidate genes with excellent haplotypes and excellent linkage disequilibrium.

Finally, function information on the promoter elements of the candidate genes was obtained from the PlantCARE website (http://bioinformatics.psb.ugent.be/webtools/plantcare/html/), and the functional effect of the SNP loci change in the promoter of the candidate genes was analyzed to reveal the causes for the increased incidence of somatic embryos.

## 3. Results

### 3.1. Screening of Extreme Materials for Somatic Embryogenesis

**Figure 1:**
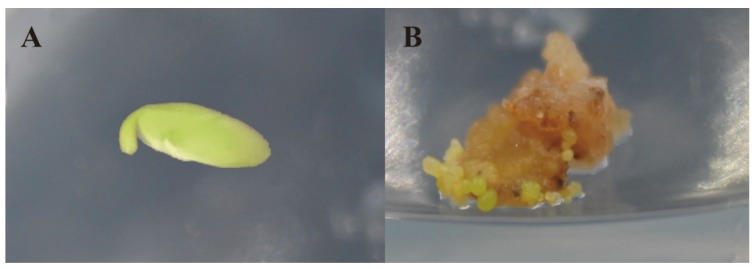
Somatic embryo culture process. (**A**) Immature embryo, before 2,4-D treatment; (**B**) Immature embryos treated with 2,4-D for 20 days.

The incidence of somatic embryos was studied in the two parents, Suinong 14 and ZYD00006, and their offspring (Figure 1). The results showed that the average incidence of somatic embryos in Suinong 14 and ZYD00006 was 10.88% and 30.27%, respectively, and the incidence of somatic embryos in the offspring of the introduced line ranged from 1% to 71.3% (Table S1).

**Figure 2:**
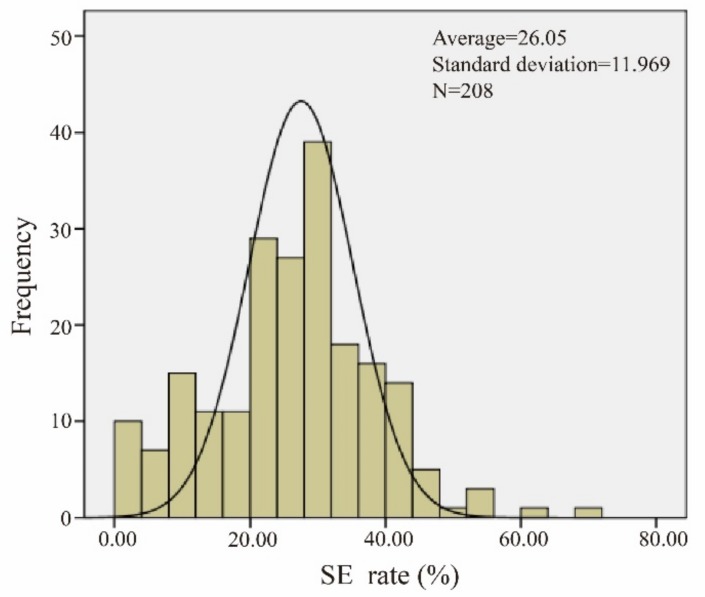
Normal distribution of somatic embryogenesis (SE). The data of the materials conform to a normal distribution.

Statistical software analysis showed that the incidence of somatic embryos in the offspring of the introduced lines was in accordance with the normal distribution (Figure 2 and Table S2). The mean of the normal distribution, plus two times the standard deviation (μ + 2σ), was used as the demarcation line for screening materials with a high somatic embryo incidence, and the mean minus two times the standard deviation (μ − 2σ) was used as the demarcation line for screening materials with a low somatic embryo incidence.

The average value of the test material was 26.05, and the standard deviation was 11.97. The demarcation line of dominant materials was found to be 49.98% by means of a double standard deviation. Five extreme dominant materials (R161, R32, R216, R156, and R203) and five extreme inferior materials (R68, R212, R207, R143, and R62) were obtained. These materials were used as extreme materials for the further study of somatic embryogenesis.

### 3.2. Analysis of the Import Fragments of Extreme Advantage CSSL Population Materials

**Figure 3:**
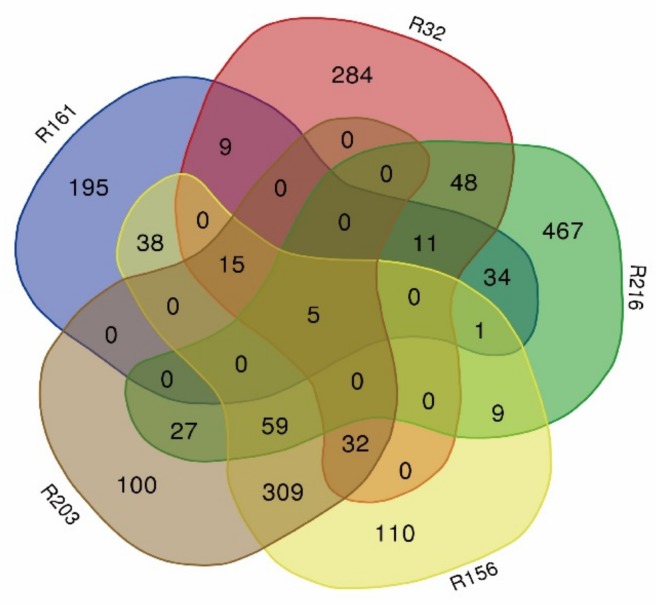
Venn diagram of the blocks in extremely dominant materials (R161, R32, R216, R156, and R203).

The introduction of the fragment blocks of five extremely dominant materials was analyzed. The material, R161, contained 308 import fragments; R32 contained 403 import fragments; R216 contained 661 import fragments; R156 contained 578 import fragments; R203 contained 547 import fragments (Figure 3).

**Figure 4:**
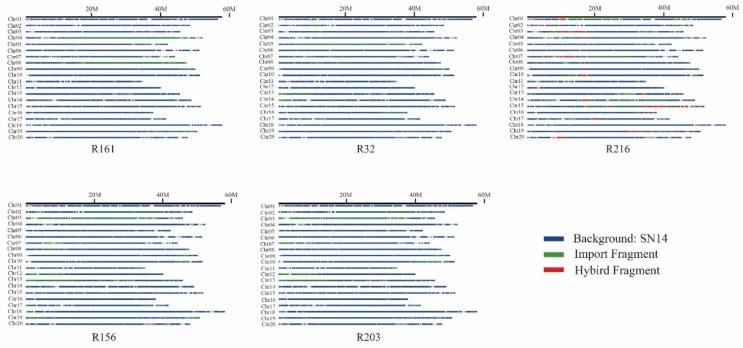
Schematic structures of the substituted segments in extremely dominant materials. Blue, green, and red represent the background SN14, import fragment, and hybrid fragment, respectively.

Among the extremely dominant materials, 123 import fragments were found in more than three, and 1281 genes were found in these blocks (Table S3). These genes were mainly concentrated on chromosomes 2, 3, 4, 6, 9, 10, 12, 13, and 19 (Figure 4).

### 3.3. Global Analysis of Transcriptome Sequencing Data

The raw data of the transcriptome sequencing material samples from the parent, Suinong 14, and 10 extreme material populations are shown in Table S3. A total of 22 samples were tested, with three replicates for each sample. The quality of sequencing is an important index for measuring the accuracy of data. After sequencing quality control (QC), chimeric read removal, raw read filtering, and low copy sequence clearance, 473.69 Gb of clean data were obtained. The amount of clean data for each sample exceeded 6 Gb. The Q20 value of all of the raw data was above 96%, and the Q30 value was above 90%. Thus, the base quality was excellent (Table S4).

Using HISAT2 software, the obtained clean reads were aligned to the soybean reference genome (*Glycine Max* wm82.a2). According to the statistics, the proportion of the clean reads of all of the sequencing materials to the number of sequenced sequences in the genome ranged from 93.05% to 96.32%. The range of reads for a uniquely mapped reference genome was 87.43%–94.22%, and for a multi-mapped reference genome, it was 1.13%–8.73%. The uniform ratio of the samples indicated that the data for the different samples were comparable. The results of the comparison were statistically significant (Table S5). The reads on the soybean reference genome were used for subsequent analysis of the expression of differential genes. The results obtained in this study were of good quality and met the sequencing criteria, which means that they could be used for subsequent analysis.

The phenotypic differences between the experimental materials could be reflected in the results of transcriptome sequencing. The transcriptome data of different materials induced by the exogenous hormone, 2,4-D, were compared and analyzed to study the expression patterns of the genes that led to differences in somatic embryogenesis. In this study, fold change ≥1 was used as a screening criterion to analyze differentially expressed genes. The results showed that 2666 differentially expressed genes were obtained. The results indicated that there were 1218 upregulated genes and 1448 downregulated genes (Table S6).

### 3.4. Analysis of Differentially Expressed Genes in Import Fragments and Transcriptome Sequencing

**Figure 5:**
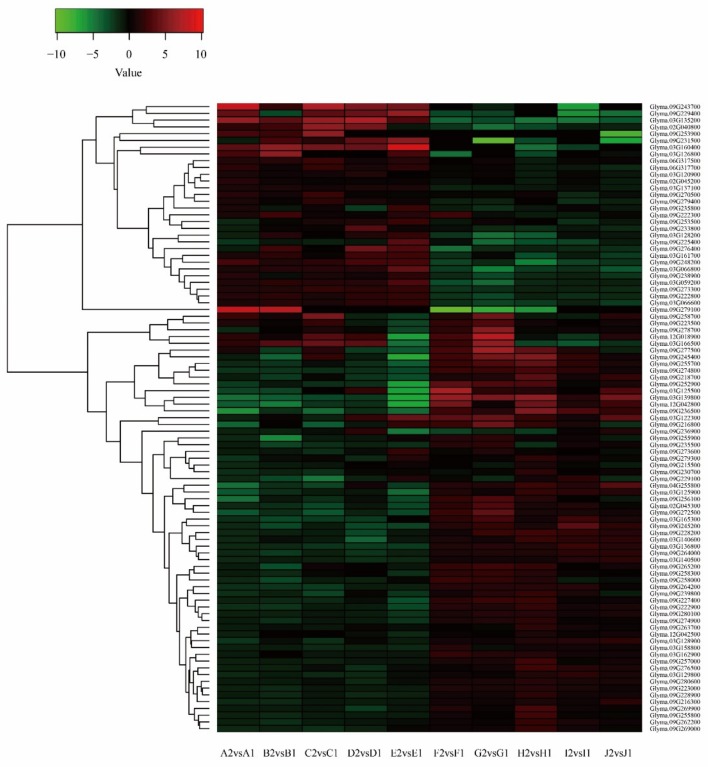
Cluster analysis of differentially expressed genes (DEGs) after combining transcriptome sequencing and import fragments.

We obtained 2666 differentially expressed genes from the transcriptome sequencing data and 1281 genes from the imported fragment data. Comparing the two groups of data, we found that there were 93 identical genes (Table S7). There were 30 upregulated genes and 63 downregulated genes. According to the relationship in the expression quantity, a heatmap of differentially expressed genes was drawn. The colors in the heatmap indicated the expression levels of the genes. The higher the expression level, the darker the color (red is upregulated, green is downregulated). Numerous differences could be observed between the dominant material group and the disadvantaged material group. In the two groups, most genes showed opposing expression patterns (Figure 5).

**Figure 6:**
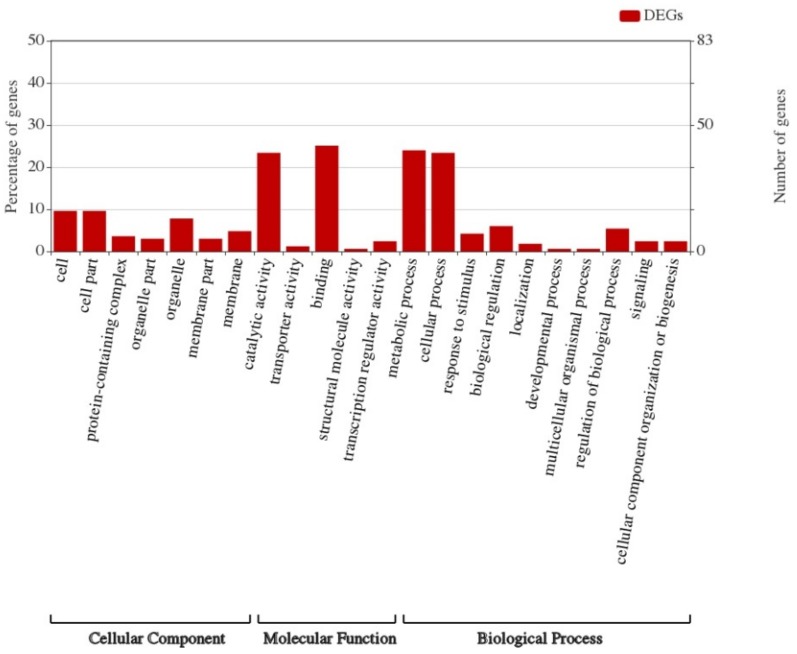
Gene Ontology analysis of transcriptome sequencing and import fragments.

Gene Ontology (GO) is widely used in the field of bioinformatics. It covers three aspects of biology: cellular components, molecular functions, and biological processes. GO enrichment analysis was performed on 167 differentially expressed genes, as shown in Figure 6. From this figure, it could be seen that the differentially expressed genes were mainly enriched in their binding-related functions of molecular functions followed by their catalytic activity, and in terms of biological processes, participating in metabolic and cellular processes was the main role of the enriched genes.

### 3.5. RT-qPCR Analysis of Candidate Genes

To verify the reliability of the transcriptome sequencing binding data in order to obtain differentially expressed genes, three genes that were possibly associated with somatic embryogenesis were selected and verified by real-time quantitative PCR (RT-qPCR): the *CLV* family gene, Glyma.03G125900 (CLAVATA3/ESR-RELATED 22), the auxin response factor, *Glyma.09G222300* (SAUR-like auxin-responsive protein family), and *Glyma.09G248200* (integrase-type DNA-binding superfamily protein), which is a gene from a family of BABY BOOM (*BBM*) genes with an AP2/ERF domain (Table S8).

These three genes had the following three characteristics: First, they were the genes contained in the import fragments of the dominant materials in the CSSL population, which were imported from the wild soybean, ZYD000006. Second, they were differentially expressed according to the transcriptome sequencing results. Third, they were genes related to cell development or somatic embryogenesis pathways.

**Figure 7:**
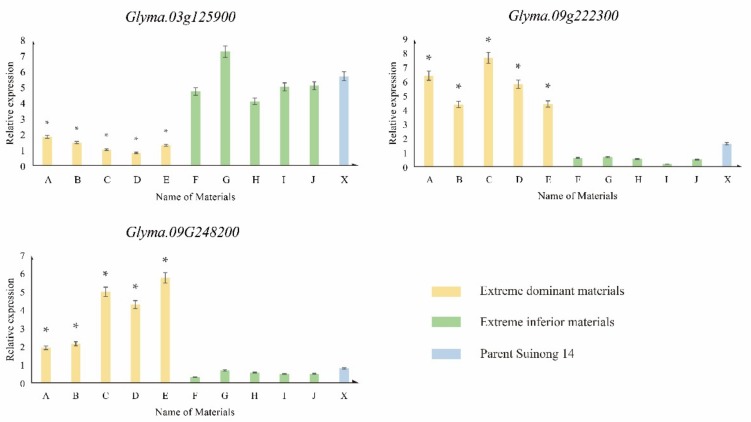
Relative expression of candidate genes by RT-qPCR. * denotes a significant difference between the extreme material and others (*p* < 0.05).

From the results, it could be seen that the relative expression of the *Glyma.03G125900* gene in materials with an extremely high incidence (A–E) was significantly decreased after 2,4-D treatment, compared with the Suinong 14 (X) control material, but this was also lower than that in materials with an extremely low incidence (F–J). The relative expression of the *Glyma.09G222300* and *Glyma.09G248200* genes in materials with an extremely high incidence (A–E) after 2,4-D treatment was significantly higher than that in the Suinong 14 (X) control materials, but it was also higher than that in materials with an extremely low incidence (F–J) (Figure 7).

The results showed that the expression patterns of those genes in transcriptome sequencing and RT-qPCR were similar, indicating that the results of transcriptome sequencing and real-time fluorescence quantitative RT-qPCR were basically the same, verifying the reliability of the data.

### 3.6. Haplotype Analysis of Candidate Genes

We selected the full-length sequences of three candidate genes and their upstream 3000 bp promoter regions in the Phytozome database as the analysis objects. DnaSP 5.10 software (University of Barcelona, Barcelona, Spain) was used to detect haplotype polymorphisms and perform a linkage disequilibrium analysis based on the phenotypic data on somatic embryo incidence in 208 CSSLs.

The phenotypic data of somatic embryo incidence in CSSL populations were calculated by SPSS 20.0. There were abundant variations in these data, which could be used for haplotype analysis.

The results showed that the differentially expressed gene, *Glyma.09G248200,* had two excellent haplotypes (the number of resources of haplotypes exceeded 5% of the total amount of CSSLs) (Table S9).

Based on the phenotypic data presented above, excellent haplotypes were analyzed by ANOVA.

**Figure 8:**
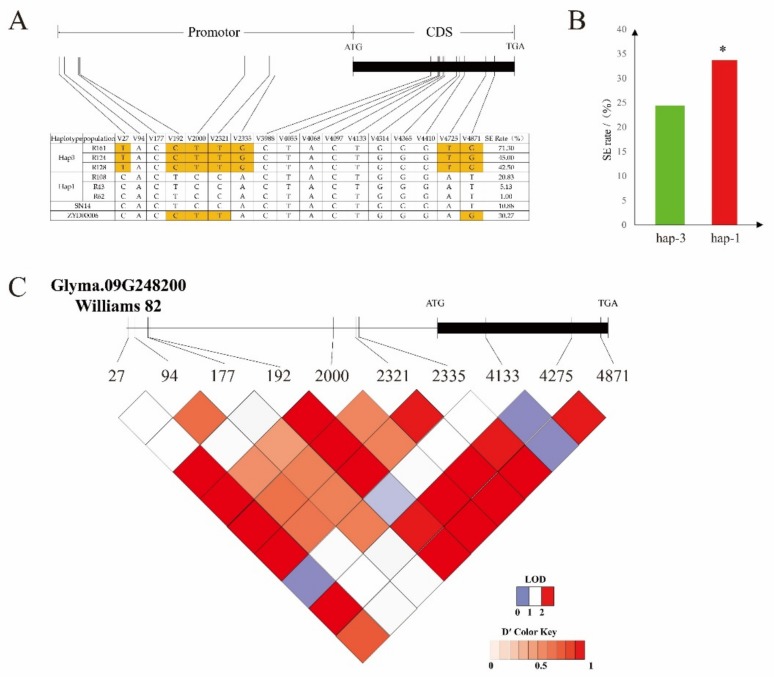
Haplotype analysis of the candidate gene. (**A**) Somatic embryogenesis rates of hap-1 and hap-3 (boxes colored in orange indicate differences between materials). (**B**) Haplotype analysis of *Glyma.09g248200* from 208 chromosome section substitution lines (CSSLs) (* denotes a significant difference between hap-3 and hap-1 (*p* < 0.05)). (**C**) Linkage disequilibrium analysis of single-nucleotide polymorphisms (SNPs), located in Glyma.09g248200. Red, from light to dark, represents the degree of linkage between SNPs.

The *Glyma.09G248200* gene has two excellent haplotypes: hap-1 and hap-3. There were significant differences in the incidence of somatic embryos (Figure 8A, Table S10).

The average incidence of the hap-1 and hap-3 somatic embryos was 24.41% and 33.71%, respectively. There were 170 hap-1 resources, accounting for 81.73% of the total population, and 14 hap-3 resources, accounting for 6.73% of the total population. The average somatic embryo incidence of hap-1 was significantly lower than that of hap-3 (Figure 8B). It is suggested that the haplotype of the gene is related to somatic embryogenesis.

SNP-27, SNP-192, SNP-2000, SNP-2321, and SNP-2335 showed single-nucleotide polymorphism (SNP) differences in the promoter region between the two haplotypes (Figure 8B), resulting in changes in the homeobox binding sites in the promoter region. SNP-4725 and SNP-4871 were the two sites in the CDS region that changed the original valine to glycine and the structure of amino acid.

According to linkage disequilibrium analysis, there was a linkage relationship between SNP-192 and SNP-2335 (Figure 8C). SNP-2335 also encoded the *cis*-acting element, CAAT-box, in the promoter and enhancer regions, which was the reason for the change in the somatic embryogenesis ability.

According to the analysis by DnaSP 5.10, *Glyma.03G125900* and *Glyma.09G222300* had only one excellent haplotype, which did not satisfy the condition for the comparative analysis of multiple haplotypes.

## 4. Discussion

It is well known that selecting a good set of materials in terms of the genetic population is very important for plant phenotype research. The CSSL population is such an ideal material. The F_1_ is generated through parental hybridization and then back-crossed many times with the recurrent parent. Through molecular marker-assisted selection technology, most of the genetic backgrounds are from recurrent parents, and a few fragments are from the experimental materials of donor parents, which reduces the interference of genetic backgrounds. This population has a good performance in the phenotypic breeding of crops and has a wide range of applications in rice [14,15,16], soybean [17], peanut [18], cotton [19], and other crops. In this study, the parent, Suinong 14, was a cultivated variety with a low somatic embryogenesis ability, and ZYD00006 was a wild soybean resource with a much higher somatic embryogenesis ability than cultivated varieties. Wild soybean contained genetic information that disappeared during the domestication of cultivated soybean. The CSSL population, constructed by the hybridization of the two strains, contained only one or several import fragments, which was helpful for us to accurately find important fragments or genes affecting the somatic embryogenesis of soybean. This population was also very important for the study of soybean somatic embryogenesis ability. The fine mapping of agronomic trait-related loci in soybean using CSSL populations would provide a theoretical basis and molecular technical support for the molecular-assisted breeding and cloning of related loci in soybean.

Studies on somatic embryogenesis using genetic populations have mainly focused on rice [20,21], wheat [22], maize [23], and soybean [4,5]. These studies have obtained several loci or molecular markers related to somatic embryogenesis. In this study, the CSSL population was sequenced. By comparing the reference genome sequences, more accurate information about loci-containing genes was obtained, making the results more accurate. At the same time, the screening of differentially expressed genes for plant-related traits by sequencing technology has become a major research method and has been reported in the field of plant somatic embryogenesis in recent years. This was the earliest method for identifying the somatic embryogenesis of *Medicago* through the cross-hybridization of cDNA [24]. Suppression subtractive hybridization (SSH) was used to identify several genes related to somatic embryogenesis in soybean [25] and cotton [26]. With the development of technology, the use of high-throughput sequencing technology improved the early detection of genes in somatic embryogenesis (SE). The Illumina digital gene expression platform was used to analyze the transcriptome data of cotton in the SE process [27]. Later, studies on *Larix leptolepis*, *Dimocarpus longan* Lour., *Cinnamomum camphora*, *Cocos nucifera* L., and *Zea mays* enriched the number of genes involved in the SE process in plants [28,29,30,31,32].

In this study, the transcriptome sequencing of extreme materials with different somatic embryogenesis abilities was carried out, and a large amount of data on differentially expressed genes was obtained. However, the large amount of data made it too difficult to find the main genes affecting the traits. Therefore, we combined the transcriptome sequencing data of differentially expressed genes with the sequence data of the CSSL population so that we could more accurately identify the key genes affecting somatic embryogenesis, which also represents the innovation of this study.

Due to the similarity between somatic embryogenesis (SE) and zygotic embryogenesis (ZE) in normal plants, the transcription factors (TFs) controlling ZE are similar to the TFs constituting SE [33]. An important feature of SE is that the embryogenesis is not limited to fertilized eggs (zygotic embryogenesis) [34]. Embryos may come from unfertilized diploid eggs (gametophytic apomixis), from maternal cells around the embryo sac (sporophytic apomixis) [35], or somatic cells (prosthesis) [36]. In addition, embryonic development can be induced by microspores/pollen grains (microspore embryogenesis) [37] and various somatic cells/tissues under appropriate in vitro conditions (somatic embryogenesis) [38].

It has also been observed that, in many cases, the expression of various regulatory genes that control cell division and differentiation can also lead to ectopic embryonic development [34]. It has been reported that the expression of at least 500 genes, including TFs, controls ZE in *Arabidopsis thaliana* [39]. Therefore, the study of differential gene expression during somatic embryogenesis is very important for the study of the SE process in soybean regeneration. As for SE regulatory genes, the addition of exogenous hormones to the in vitro cultures of different plants leads to the excessive induction of TF expression, which results in the formation of somatic embryos in tissues. This result reflects the important role that exogenous hormones play in changing the fate of plant development [40,41]. In *Arabidopsis thaliana*, 43% of the TF genes regulating SE have been annotated as hormone-related [42]. In addition to the TFs involved in auxin metabolism and signal transduction, genes related to cytokinin (CK), abscisic acid (ABA), jasmonic acid (JA), ethylene (ET), gibberellin (GA), and brassinosteroid (BR) have been reported in *Arabidopsis* and other plants [42,43,44,45,46].

Among the three candidate genes identified in this study, the CLV family gene, *Glyma.03G125900* (CLAVATA3/ESR-RELATED 22), is an important switch of WUS, which regulates embryogenesis [47,48]. It has been found that CLV family genes are transcribed in one or more tissues during development, indicating that they encode functional products, are widely expressed in many tissues, and they may act as secretory signaling molecules in different pathways of growth and development [49].

*Glyma.09G222300* (SAUR-like auxin-responsive protein family), a member of the transcription-mediated auxin signaling pathway, affects plant growth and development by regulating cell division, elongation, and differentiation [50]. Aux/IAA, GH3, SAUR, and other auxin-responsive genes are mainly involved in the regulation of plant growth and development [51,52]. Studies have shown that the root and leaf growth of SAUR-expressing plants in *Arabidopsis thaliana* are more vigorous, suggesting that SAUR-expressing plants may participate in cell division and affect development [53].

*Glyma.09G248200* (integrase-type DNA-binding superfamily protein) gene is a BABY BOOM (BBM) gene with an AP2/ERF domain. The gene is expressed in the early division of tissues and plays a central role in different developmental processes, including embryogenesis. Studies on *Arabidopsis* have shown that BBM promoter activity is largely confined to mitotic cells, which then develop into somatic embryos and calluses. In some explants that did not participate in morphogenesis, BBM promoter activity was absent or almost invisible. Therefore, the expression of BBM in vitro is closely related to cell proliferation and morphogenesis [54].

In addition to auxin, the BBM gene is reported to be associated with ABA, GA, and JA signal transduction. Therefore, the BBM gene is assumed to integrate multiple hormone inputs in plant development and act as a “network hub” [55,56,57,58,59,60,61].

Haploview is software for haplotype analysis. By comparing the whole genome sequence information with the genome sequence information of a natural population, Haploview can find mutation sites related to target traits, which is very important for the next step in gene function verification. Its successful application in rice [62], wheat [63], cherry [64], *Arabidopsis* [65], and other species has resulted in the genotyping and markers of related traits [66,67,68]. In this study, we carried out a haplotype analysis of the differentially expressed genes of target traits in a CSSL population, obtained excellent haplotype materials for somatic embryogenesis, analyzed the differences between the SNP loci of gene promoters, and predicted possible changes in the binding sites of promoter regions and how they might relate to the observed phenotypic differences in materials.

In summary, we identified important genes that affect somatic embryogenesis by combining transcriptomic analysis of groups and the CSSL population. We found three candidate genes related to somatic embryogenesis. The possible mechanism of these differentially expressed genes was discussed from the perspective of haplotype analysis. However, further studies are needed to elucidate the molecular mechanism of these differentially expressed genes.

## Supplementary Materials

The following are available online at https://www.mdpi.com/2073-4425/10/11/943/s1. Table S1: Somatic Embryos in Materials. Table S2: Phenotypic Statistics of SE (%). Table S3: Gene List in the Block. Table S4: Data Table. Table S5: Map Stat. Table S6: DEG of Transcriptome Sequencing. Table S7: Information of Identical Genes from Two Compered Groups. Table S8: Information of Candidate Genes. Table S9: Candidate Gene Haplotype in CSSL. Table S10: Phenotypic Differences.
